# First record of the complete mitochondrial genome of the mantis shrimp, *Gonodactylaceus randalli* (Manning, 1978) (Stomatopoda: Gonodactylidae)

**DOI:** 10.1080/23802359.2021.1872441

**Published:** 2021-02-11

**Authors:** Hee-seung Hwang, Jongwoo Jung

**Affiliations:** a Research Institute of EcoScience, Ewha Womans University, Seoul, South Korea; b The Division of EcoCreative, Ewha Womans University, Seoul, South Korea; c Department of Science Education, Ewha Womans University, Seoul, South Korea

**Keywords:** Crustacea, *Gonodactylaceus randalli*, mitochondrial DNA, mitochondrial genome, Stomatopoda

## Abstract

Biofouling has long been known as a major route for the invasion by non-indigenous species. The mantis shrimp, *Gonodactylaceus randalli,* is the first stomatopod species that has been identified in a biofouling community. In this study, we sequenced and analyzed the complete mitochondrial genome sequence of this species for the first time. Its mitochondrial genome is 15,907 bp in length and comprises 13 protein-coding genes, 22 transfer RNA genes, 2 ribosomal RNA genes, and a non-coding A + T-rich region. The overall base composition in the heavy strand is as follows: A: 37.3%, T: 31.3%, G: 11.9%, and C: 19.4%, with a G + C content of 31.3%. The phylogenetic analysis revealed that *G*. *randalli* belonged to the families Protosquillidae, Gonodactylidae, and Takuidae, in the same clade, within the superfamily Gonodactyloidea. This is the first record of the complete mitochondrial genome sequence of the genus *Gonodactylaceus*.

Biological invasions by non-indigenous species (NIS) are known to negatively affect biodiversity and ecosystems, human and wildlife health, and the economy (Wilcove et al. 1998; Daszak et al. 2000; Pimentel et al. 2005; Lodge et al. 2006). In order to address this issue, several studies have been conducted on invasion pathways. For instance, one study assessed a semisubmersible oil platform as a potentially major vector for biofouling-mediated invasion. Among the crustaceans identified in that study, *Gonodactylaceus randalli* is the first stomatopod species that has been identified in a biofouling community (Yeo et al. 2010). With the aim to provide preliminary data to support the preparation against biological invasions, including biofouling, we hereby present the first complete mitochondrial genome sequence of *G. randalli* (Manning, 1978).

Specimen of *G. randalli* was collected from the subtidal zone of Bohol Island, the Philippines (9°58′42.4″N, 124°00′52.0″E), on 11 February 2019. A voucher specimen was preserved in 80% ethyl alcohol and was deposited at the Research Institute of EcoScience, Ewha Womans University, Seoul, South Korea (Registration number: EWNHMAR770). Total genomic DNA was extracted from the leg muscle tissue using the MGIEasy DNA Library Prep Set (MGI, Shenzhen, China). Whole genome sequencing was performed with the MGISEQ-2000 platform (MGI, Shenzhen, China). MITObim (Hahn et al. 2013) was used for the assembly of the complete mitochondrial genome, which was then annotated with MITOS (Bernt et al. 2013).

The complete mitochondrial genome of *G. randalli* comprises 15,907 bp and encodes 13 proteins, 22 transfer RNAs, and two ribosomal RNAs with a putative control region. For the protein-coding genes, the most commonly shared start codon is ATG (i.e., in *COX2*, *COX3*, *NAD3*, *NAD4*, *NAD4L*, and *CYB*). In the case of *COX1*, the start codon is ACG, as often observed in the malacostracan mitochondrial DNA (Liu and Cui 2010). The start codon of *NAD1* and *NAD5* is ATA, while that of *ATP8* is ATC. As for *NAD2* and *NAD6,* the start codon is ATT. Furthermore, the most common termination codon is TAA. However, the termination codon of *NAD1* and *CYB* is TAG, while those of *COX2*, *NAD3*, and *NAD6* are AAT, ATT, and CAT, respectively. Notably, the respective termination codons of *COX2*, *NAD3*, and *NAD6* are incomplete. Similar cases also occur with several protein-coding genes in the complete mitochondrial genomes of stomatopods available to date, and are due to excessive polyadenylation (Ojala et al. 1980, 1981). The overall base composition of the mitochondrial genome of *G*. *randalli* was A: 37.3%, T: 31.3%, G: 11.9%, and C: 19.4%, with a G + C content of 31.3%. The lengths of the *LrRNA* and *SrRNA* genes are of 1,354 bp and 822 bp, respectively. The length of the 22 *tRNA*s was identified to range from 62 to 79 nucleotides. Finally, the putative control region is located between the *SrRNA* and *tRNA-Ile* and is 1,010 bp in length.

After alignment with Clustal W (Thompson et al. 2003), the molecular phylogenetic tree was constructed based on the concatenated sequences of 13 protein-coding genes with the maximum likelihood, using MEGA X (Kumar et al. 2018). The GTR + G + I model was identified as the best-fit model, using the ModelFinder 54 (Kalyaanamoorthy et al. 2017) with a bootstrap of 1,000 replicates.

To explore the phylogenetic position of *G*. *randalli*, five squilloid and three gonodactyloid species were compared with *G*. *randalli* (MW019425). Based on the mitogenome sequences previously deposited in GenBank and using a lysiosquilloid species as an outgroup, the analysis revealed that *G*. *randalli* belonged to the genera *Chorisquilla* (Protosquillidae), *Gonodactylus* (Gonodactylidae), and *Taku* (Takuidae), in the same clade, within the superfamily Gonodactyloidea. Notably, *Gonodactylaceus randalli* and *Gonodactylus chiragra*, two species belonging to the same family, are more closely related to protosquillid and takuid species, respectively, than to each other. Further taxonomic studies of this superfamily are required (Figure 1).

**Figure 1.**
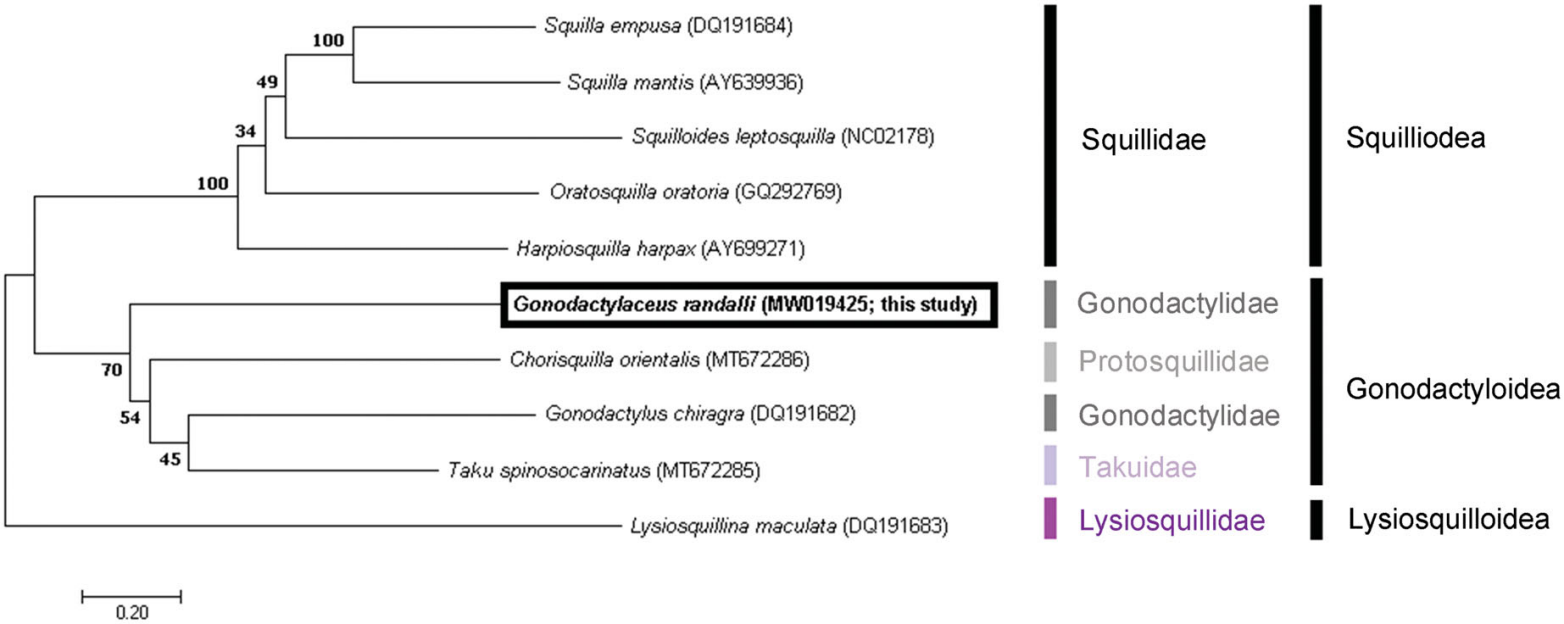
Phylogenetic tree of the complete mitochondrial genomes from ten stomatopods: *Squilla empusa* (DQ191684), *Squilla mantis* (AY639936), *Squilloides leptosquilla* (NC02178), *Oratosquilla oratoria* (GQ292769), *Harpiosquilla harpax* (AY699271), *Chorisquilla orientalis* (MT672286), *Taku spinosocarinatus* (MT672285), *Lysiosquillina maculata* (DQ191683), *Gonodactylus chiragra* (DQ191682), and *Gonodactylaceus randalli* (MW019425). The tree was constructed by using the maximum likelihood method.

This is the first record of the complete mitogenome sequence of a stomatopod that is member of a biofouling community. Furthermore, it is the first record of a complete mitogenome sequence in the genus *Gonodactylaceus.* Along with our results, further mitogenomic analyses of undetermined taxa within stomatopods would improve our understanding of their taxonomic relationship and phylogeny.

## Data Availability

The genome sequence data that support the findings of this study are openly available in GenBank (National Center for Biotechnology Information) at https://www.ncbi.nlm.nih.gov, accession no. MW019425. The associated BioProject, SRA, and Bio Sample numbers are PRJNA663697, SRR12929238, and SAMN16176943, respectively. The data that support the findings of this study are also openly available in Mendeley Data at https://data.mendeley.com/datasets/7jr5gychkn/1.
